# Meningoencephalitis associated with GAD65 autoimmunity

**DOI:** 10.3389/fimmu.2023.1120894

**Published:** 2023-03-09

**Authors:** Zuying Kuang, José Fidel Baizabal-Carvallo, Mohammad Mofatteh, Sifen Xie, Mengqiu Pan, Jinlong Ye, Lihua Zhou, Shuiquang Yang, Zhanhang Wang, Yimin Chen, Yaqin Li

**Affiliations:** ^1^ Department of Neurology, GuangDong 999 Brain Hospital, Guangzhou, China; ^2^ Parkinson’s Disease Center and Movement Disorders Clinic, Department of Neurology, Baylor College of Medicine, Houston, TX, United States; ^3^ Department of Sciences and Engineering, University of Guanajuato, León, Mexico; ^4^ School of Medicine, Dentistry and Biomedical Sciences, Queen’s University Belfast, Belfast, United Kingdom; ^5^ Department of Neurology, Foshan Sanshui District People’s Hospital, Foshan, Guangdong, China; ^6^ Department of Neurology, The Seventh Affiliated Hospital, Sun Yat-sen University, Shenzhen, Guangdong, China

**Keywords:** anti-glutamic acid decarboxylase, antibodies, encephalitis, autoimmunity, GAD65, meningoencephalitis

## Abstract

**Background:**

Encephalitis has been recognized in patients with autoimmunity related to the 65-kDa isoform of glutamic acid decarboxylase (GAD65) antibodies; however, patients with meningoencephalitis associated with those antibodies have been rarely identified in the medical literature. We aimed to define the frequency, clinical features, response to therapy, and functional outcomes of patients with meningoencephalitis associated with GAD antibodies.

**Methods:**

We retrospectively studied consecutive patients attending a tertiary care center for evaluation of an autoimmune neurological disorder from January 2018 to June 2022. The modified Rankin Scale (mRS) was used to assess the functional outcome at the last follow-up.

**Results:**

We evaluated 482 patients with confirmed autoimmune encephalitis during the study period. Four among the 25 patients with encephalitis related to GAD65 antibodies were identified. One patient was excluded owing to the coexistence of NMDAR antibodies. Three male patients aged 36, 24, and 16 years had an acute (*n* = 1) or subacute (*n* = 2) onset of confusion, psychosis, cognitive symptoms, seizures, or tremor. No patient had fever or clinical signs of meningeal irritation. Mild pleocytosis (<100 leukocytes/106) was identified in two patients, whereas one patient had normal CSF. Following immunotherapy with corticosteroids (*n* = 3) or intravenous immunoglobulin (*n* = 1), significant improvement was observed in all three cases, achieving a good outcome (mRS 1) in all cases.

**Conclusion:**

Meningoencephalitis is an uncommon presentation of GAD65 autoimmunity. Patients present with signs of encephalitis but with meningeal enhancement and have good outcomes.

## Introduction

The knowledge of autoimmunity related to the 65-kDa isoform of glutamic acid decarboxylase (GAD65), the enzyme that converts the excitatory glutamate to inhibitory gamma-aminobutyric acid (GABA), has markedly evolved in recent decades (1). Cerebellar ataxia, epilepsy, and stiff-person syndrome were clinical syndromes initially linked to the presence of these antibodies (Abs) (2, 3). However, in these cases, conspicuous brain or spinal cord inflammation is usually absent in neuroimaging studies. More recently, a small proportion of patients with GAD65 autoimmunity have been described with clinical and radiological signs of brain inflammation that may affect the limbic and/or extralimbic cortices (4). Parenchymal inflammation in such cases is believed to originate from an autoimmune cellular response, rather than a direct effect of GAD65 Abs.

Aseptic meningitis is characterized by the lack of an identifiable bacterial cause in cerebrospinal fluid (CSF) cultures (5). Viruses are the most common cause of aseptic meningitis (6). Among them, enteroviruses and varicella zoster virus are the most common (6). Selected patients with inflammatory or autoimmune diseases, including Still disease, systemic lupus erythematosus (SLE), sarcoidosis, Behçet’s disease, Sjögren’s syndrome, and periodic fever syndrome, have been identified with aseptic meningitis secondary to the underlying systemic disorder (5–10). In contrast, meningeal enhancement has been identified in patients with paraneoplastic or autoimmune encephalitis, specifically anti-*N*-methyl-d-aspartate receptor (NMDAR) encephalitis (11, 12). However, neuroimaging findings consistent with meningeal enhancement are rarely seen in patients with GAD65 autoimmunity.

In this study, we aimed to retrospectively characterize the frequency, clinical findings, response to therapy, and functional outcomes in patients with meningoencephalitis associated with GAD Abs. As patients with GAD65 encephalitis frequently present with inflammatory CSF, possibly reflecting meningeal inflammation, but without contrast enhancement on MRI, hence, we defined “meningoencephalitis” by the presence of patchy or diffuse contrast enhancement in the leptomeninges, supporting an inflammatory process disrupting the blood–brain barrier in these sites, with or without clinical or neuroimaging findings of brain parenchyma inflammation.

## Methods

We retrospectively studied consecutive patients referred for evaluation at the Department of Neurology and Immunology of the GuangDong 999 Brain Hospital in Guangdong Province in the People’s Republic of China, a tertiary care center for autoimmune neurological disorders from January 2018 to June 2022. Patients were enrolled if they had clinical and neuroimaging features of acute or subacute encephalitis attributed primarily to anti-GAD65 autoimmunity, owing to the presence of serum anti-GAD65 Abs and/or positive anti-GAD65 Abs in the CSF (13).

### General assessment and follow-up

All patients underwent general and neurological examinations to assess for rheumatological or systemic inflammatory causes of meningitis. History of medication, illicit drug consumption, or poisoning was determined. Blood count, glucose, kidney, and liver function tests were performed to assess for evidence of metabolic derangement. The outcome was defined according to the modified Rankin Scale (mRS) determined at the last date of follow-up. The mRS scores were categorized as follows: 0, no symptoms; 1, total independence despite symptoms; 2, unable to carry all previous activities but look after own affairs; 3, requiring some help but able to walk without assistance; 4, unable to walk without assistance; 5, bedridden; and 6, death. A favorable outcome was defined as an mRS score between 0 and 2, while a poor outcome was defined as an mRS score of 3-5 at the last follow-up.

### Antibody screening

We used commercially available cell-based assay or radioimmunoassay to check for anti-GAD65 Abs. Antibody screening ruled out anti-glial fibrillary acidic protein (GFAP), anti-NMDAR, anti-LGI1, anti-CASPR2, anti-GABA_B_, anti-GABA_A_, anti-GlyR, anti-AMPA, anti-DPPX, anti-DRD2, anti-IgLON5, anti-mGlutR1, anti-mGlutR5, anti-MOG, and anti-Neurexin-3.

### Infectious disease screening

All patients underwent extensive diagnostic tests to rule out infectious causes. A lumbar puncture was performed in four patients and CSF was collected. Opening pressure was measured in all cases in the lateral decubitus position, with the legs and neck in a neutral position. Intracranial hypertension was considered in case the opening pressure was above 200 mmH_2_O, whereas intracranial hypotension was considered in case the opening pressure was below 60 mmH_2_O (14, 15). CSF samples were extensively assessed for white blood cells (WBC), proteins, and glucose levels using bacterial cultures and Gram stain. Acid-fast staining and India ink/cryptococcal antigen preparation were done in order to detect tuberculosis or *Cryptococcus neoformans*, respectively (16). Polymerase chain reaction was carried out in the CSF to assess for the presence of viral causes of meningitis. Hepatitis B (HBV), hepatitis C (HCV), and human immunodeficiency virus (HIV) type 1 and 2 serology were carried out in these patients.

## Results

Among the 482 patients evaluated with confirmed autoimmune encephalitis during the study period, there were 25 patients with encephalitis associated with positive anti-GAD65 Abs. Four (16%) of such patients had meningeal enhancement identified in the brain MRI. However, one patient, a 9-year-old girl with subacute headache and positive serum GAD65 Abs, was excluded from the study, owing to the presence of anti-NMDAR Abs in the CSF. The remaining three were all male patients, of Chinese origin, and between 16 and 36 years of age (**Table 1**). Extensive diagnostic studies ruled out metabolic, infectious, or drug-induced meningitis in all cases.

**Table 1:** Summary of clinical and demographic features of GAD65 patients with meningeal enhancement.

Age/sex (Author)	Clinical manifestations	MRI	EEG	Therapy	Response to therapy
36/M (This report)	Behavioral changes, confusion, poor sleep, visual hallucinations	Diffuse leptomeningeal enhancement	Slow waves in bilateral frontal lobes and centrotemporal regions	Risperidone MTP 40 mg/day with progressive dose reduction	Complete recovery (mRS: 0) at 16 months
24/M (This report)	Behavioral changes, severe throbbing headache, delusional thoughts, cognitive disturbances, motor/language perseverance, insomnia	Diffuse leptomeningeal enhancement, thickening of the tentorium	Diffuse slow waves; epileptiform discharges in the bilateral frontal lobes and right temporal lobe	Olanzapine IVIg 20 g/day for 5 days MTP 40 mg/day with progressive dose reduction	Prominent recovery (mRS: 1) at 12 months
16/M (This report)	Sleepiness, fatigue, insomnia	Scattered line-like enhancement in intracranial sulci	N/A	IVIg 25 g/day for 5 days MTP 500 mg/day for 5 days, followed by prednisone with progressive dose reduction	Complete recovery (mRS: 0) at 10 months
21/F (Triplett 2018)	Seizures, coma	Leptomeningeal enhancement; bilateral frontal, insular, and temporal hyperintensities	Generalized slowing, left frontal epileptic activity	Antiepileptics; cyclophosphamide, rituximab, IVIg, MTP, mycophenolate mofetil, prednisone	Mild improvement
44/F (Salari 2022)	Cognitive symptoms and mental confusion	Hydrocephalus, meningeal enhancement^a^	N/A	MTP	Good recovery

F, female; IVIg, intravenous immunoglobulin; M, male; MTP, methylprednisolone; mRS, modified Rankin Scale; N/A, not available.

**^a^** Not observed in published neuroimaging.

### Case 1

**Figure 1:**
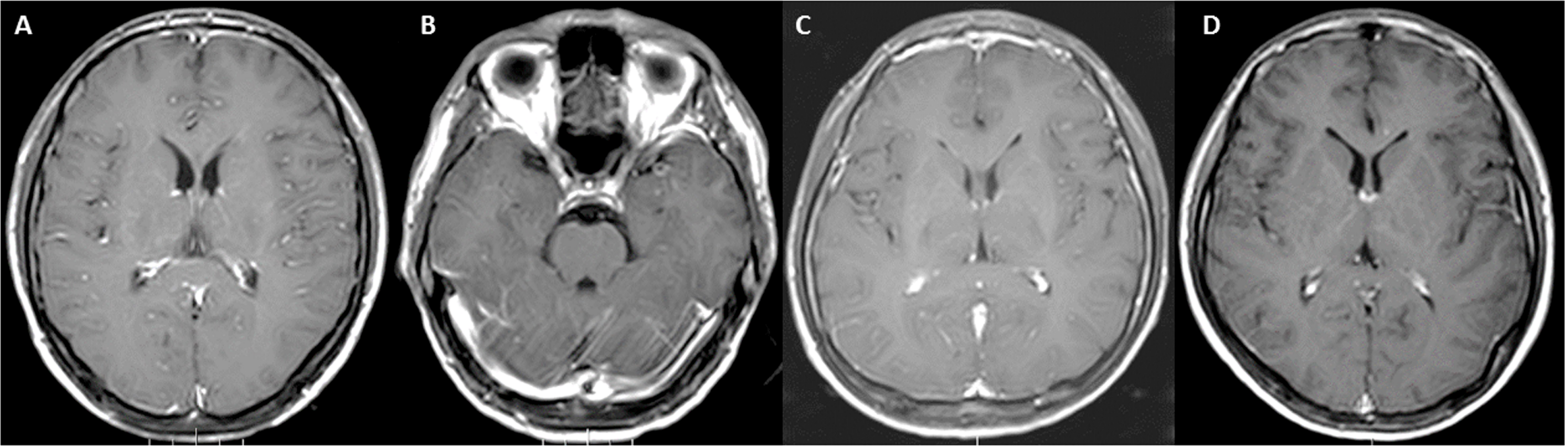
Contrast -enhanced brain MRIs of **(A, B)** case 1 show diffuse enhancement of the leptomeninges without abnormalities in the brain parenchyma. **(C)** Case 2 and **(D)** case 3 also show conspicuous and scattered enhancement, respectively of the leptomeninges.

A 36-year-old male patient presented for evaluation of a 3-month history of behavioral changes and poor sleep. There was no history of fever or seizures. The patient presented with episodes of odd behavior, restlessness, irritability, aggressiveness, and agitation. There were sporadic visual hallucinations. Neurological examination was remarkable for fluctuating mental confusion with disorientation and bilateral upper limb resting tremor. There was no focal paralysis. Nuchal rigidity and Kernig’s and Brudzinski’s signs were all negative. Brain MRI showed diffuse leptomeningeal enhancement with scattered non-enhancing hyperintensities in the white matter (**Figures 1A, B**). EEG showed diffuse slow waves in the bilateral frontal lobes and bilateral centrotemporal regions, mostly on the right side. Lumbar puncture revealed an opening pressure of 150 mmH_2_O, and the CSF showed elevated WBC, 39 × 10^6^/L (normal ≤5 × 10^6^/L); lymphocytes, 74%; proteins, 0.28 g/L (normal: 0.15-0.45 g/L); and glucose, 3.4 mmol/L (range: 2.5-4 mmol/L). The patient had positive GAD65 Abs in the serum 1:10, but these Abs were negative in the CSF. Investigations for infectious causes were all negative. During the evaluation, the patient was diagnosed with type 2 diabetes mellitus with glycosylated hemoglobin (HbA1c) (8.6%) and received treatment with oral repaglinide and metformin. Treatment with progressively higher doses of risperidone did not provide benefit. However, marked clinical improvement was observed following treatment with oral methylprednisolone 40 mg per day for 2 weeks followed by progressively decreasing doses of 4 mg every week. The patient achieved an mRS score of 0 at the 16-month follow-up, after the onset of symptoms.

### Case 2

A 24-year-old male patient came for evaluation of sudden onset of abnormal behavior and headache that started a few hours before the presentation. There was no history of a triggering event including poisoning or drug consumption. The patient and family denied fever or seizures. The clinical picture was characterized by severe, generalized, throbbing headache plus abnormal behavior with episodes of irritability, motor and language perseverance, disorientation, poor concentration, altered memory, and judgment. There were delusional thoughts, and the patient complained of being electrocuted, although no historical or clinical evidence of such an event was identified. Ritualistic behavior such as repetitive knocking on the wall was also present. There was evidence of insomnia and moderate anxiety. The neurological examination did not show a focal deficit; meningeal and cerebellar signs were negative. Cranial nerves were normal. Brain MRI showed extensive meningeal enhancement with a thickening of the tentorium more prominent on the right side. Brain parenchyma did not show abnormal hyperintensities or contrast enhancement (**Figure 1C**). EEG showed diffuse slow waves; epileptiform discharges were identified in the bilateral frontal lobes and right temporal lobe. Lumbar puncture revealed an opening pressure of 120 mmH_2_O, and cell count, proteins, and glucose levels were all normal in the CSF. Anti-GAD65 Abs were positive in the serum (1:30) and cerebrospinal fluid (1:10). The patient initially received variable doses of oral olanzapine with partial improvement. This was followed by a course of intravenous immunoglobulin (IVIg) 20 g per day for 5 days (total: 100 g) plus oral methylprednisolone 40 mg per day for 2 weeks followed by progressively decreasing doses of 4 mg every week. The patient achieved an mRS of 1 at the 12-month follow-up, after the onset of symptoms with a sporadic headache.

### Case 3

A 16-year-old male patient presented for evaluation of a 20-day history of severe daytime sleepiness. There were no apparent precipitating events, and no recent history of fever, psychosis, mental confusion, behavioral changes, or seizures was recorded. The patient showed an increased propensity to fall asleep, poor responsiveness during episodes of sleeping, generalized fatigue, and night insomnia with sleep fragmentation. The neurological examination was consistent with normal cranial nerves. Muscle strength and tendinous reflexes were also normal. Babinski sign was negative, and there were no signs of meningeal irritation. Brain MRI showed a scattered line-like enhancement in the intracranial sulci. The right tentorium was thickened and showed contrast enhancement (**Figure 1D**). The lumbar puncture showed an opening pressure of 100 mmH_2_O, and the CSF showed elevated WBC, 18 × 10^6^/L (normal ≤5 × 10^6^/L); lymphocytes, 70%; mildly elevated proteins, 1.14 g/L (normal: 0.15-0.45 g/L); and glucose, 3.2 mmol/L (range: 2.5-4 mmol/L). Anti-GAD65 Abs were positive in the serum (1:10), confirmed with radioimmunoassay with >2,000 U/ml, and in the cerebrospinal fluid (1:10). The patient received treatment with IVIg 25 g/day for 5 days and methylprednisolone 500 mg per day for 5 days followed by oral prednisone with progressively decreasing doses. The patient achieved an mRS score of 0 at the 10-month follow-up, after the onset of symptoms.

## Discussion

In this study, we reported three patients with clinical–radiological findings consistent with meningoencephalitis. All three were male patients, despite the higher frequency of GAD65 autoimmunity in women (4). Reports of meningeal involvement defined as contrast enhancement in the leptomeninges in patients with GAD65 autoimmunity are very scarce. A 44-year- old female patient has been reported under the diagnosis of “meningoencephalitis” associated with GAD65 Abs, 1 month after receiving remdesivir for COVID-19 infection (**Table 1**). The CT scan showed hydrocephalus and some meningeal enhancement; however, it is unclear whether the findings were related to COVID-19 infection (17). On the other hand, Triplett and colleagues reported on a 21-year-old female patient with seizures and progressively decreased level of consciousness with MR showing leptomeningeal enhancement aside from prominent hyperintense lesions involving both frontal lobes (18). The patient showed clinical worsening despite the use of first-line immunosuppressive drugs but responded to cyclophosphamide and rituximab (18).

The question is whether our patients actually had a neurological disorder secondary to GAD65 Abs, as they had a relatively low titer of Abs. Case 3 had >2,000 U/ml in the serum by RIA. This is consistent with GAD65 Abs titers that are considered high according to Saiz and colleagues (13). It is possible that GAD65 Abs 1:10 reported in commercially available cell-based assay are consistent with titers over 2,000 U/ml by RIA. Low titers of GAD65 Abs have been detected in up to 1% of the general population and 5% with various neurological disorders (19); therefore, these Abs may coincide and be confused as the cause of a specific neurological disorder. For example, patients with some forms of viral meningitis (i.e., related to the enterovirus) or rheumatic disorders may have meningeal enhancement with a benign clinical course and response to corticosteroids. The suspected autoimmune basis in our patients is supported by the lack of identification of specific infectious agents, despite extensive evaluation, the absence of recent history of poisoning or drug consumption, and no identification of metabolic or rheumatic diseases in the follow-up. Moreover, there is emerging evidence that low titers of GAD65 Abs may be related to some neurological syndromes, such as cerebellar ataxia (3).

The pathological role of GAD65 Abs has been questioned, mostly as the target antigen is intracellular. In contrast, the role of cellular autoimmunity has been highlighted. There are few pathological reports in patients with GAD65 autoimmunity showing microglial proliferation and mild infiltration of CD8^+^ cytotoxic T cells in the anterior horn cells of the spinal cord in a patient with stiff-person syndrome (20). On the other hand, in a pathological study of three cases with limbic encephalitis related to GAD65 encephalitis, parenchymal infiltration of CD3^+^ T cells was low but higher than that of the controls, with intermediate infiltration of CD8^+^ T cells between patients with autoimmunity related to onconeural and surface antigen Abs (21). Apposition of GrB^+^ lymphocytes to single neurons and CD107a, a lysosomal-associated membrane protein-1, both markers of cytotoxic cell attack, were identified in a single patient with limbic encephalitis associated with GAD Abs (21). These findings were coupled with the loss of neural tissue but glial preservation, suggesting that a cytotoxic rather than a humoral response underlies the pathogenesis in these patients. Moreover, the ratio of CD8/CD3 in the perivascular space of blood vessels is lower than in the parenchyma, supporting the CD8^+^ T-cell migration into the brain parenchyma (21). We speculate that a local cellular inflammatory response in the leptomeninges may occur disrupting the blood–brain barrier leading to contrast enhancement. This is supported by the presence of CD3^+^ T cells in the leptomeninges in a patient with GAD65 meningoencephalitis (18).

Meningeal enhancement has been found in patients with anti-NMDAR encephalitis, which is the prototype of autoimmune encephalitis. In these patients, an abnormal MRI is observed in less than 50% of cases (11). However, among patients with abnormal MRIs, medial temporal and frontal hyperintensities in T2W and FLAIR sequences along with leptomeningeal enhancement are the most common findings (11). Evidence from cytokine dynamics suggests that patients with anti-NMDAR encephalitis have an early chemoattractant immune response despite the paucity of cellular T- and B-cell response identified in the brain parenchyma in most of these patients (22). Minimal inflammatory infiltrates in the leptomeninges have been identified in patients with anti-NMDAR encephalitis (23). It is unclear why patients with GAD65 encephalitis uncommonly present with meningeal enhancement. Similar to anti-NMDAR encephalitis, patients with GAD65 encephalitis may show a paucity of inflammatory T-cell infiltrates in the leptomeninges that appears during a specific time window during the evolution; however, further studies are required to confirm these findings.

None of our patients had positive anti-GFAP Abs; patients with autoimmunity associated with these Abs have a median age at onset of 44 years and are most commonly women (54%) (24). Perivascular radial enhancement perpendicular to the ventricles is one characteristic, and it has been detected in about half of the cases in some series; meningeal enhancement is also a distinctive feature (25). Patients with anti-GFAP Abs usually have a high number of cells in the CSF (>50 × 10^6^/L), which contrasts with the paucity of inflammatory response found in the CSF of patients with GAD65 autoimmunity. Moreover, a third of cases may have an underlying neoplasm (24), contrasting with the apparent low occurrence of cancer in patients with GAD65 autoimmunity.

The main limitation of our study is the lack of pathological specimens in order to assess the presence of lymphocyte infiltration in the meninges. However, justification of leptomeningeal biopsy may be questionable in a patient with aseptic meningitis showing rapid recovery with anti-inflammatory therapies. In this regard, the identification of CSF cytokines can be helpful in understanding the immunological dynamics of T and B cells, which may yield the type of immunological response in these patients.

## Conclusion

In summary, a clinical picture characterized by the presence of signs of encephalitis combined with meningeal enhancement in the brain MRI but lack of fever and/or signs of meningeal irritation may be observed in patients with otherwise positive GAD65 Abs. These patients showed a rapid response to immunotherapy with a favorable outcome. Further studies should clarify the role of GAD65 autoimmunity in meningeal inflammation.

## Data availability statement

The original contributions presented in the study are included in the article/supplementary material. Further inquiries can be directed to the corresponding authors.

## Ethics statement

The studies involving human participants were reviewed and approved by the Guangdong999 Brain Hospital Institute Review Board. Written informed consent for participation was not required for this study in accordance with the national legislation and the institutional requirements. Written informed consent was not obtained from the individual(s) for the publication of any potentially identifiable images or data included in this article.

## Author contributions

YC, JB-C, ZK, ZW, and YL designed the study and drafted the manuscript. All authors reviewed and approved the final manuscript.
